# The Persistence of High Maternal Anaemia and Low Birthweight Despite Declining Malaria Transmission and Prevalence in Ghana

**DOI:** 10.3390/pathogens15060618

**Published:** 2026-06-09

**Authors:** Harry Tagbor, Joseph Osarfo, Doris Okyere, Ekoue Kouevidjin, Matilda Aberese-Ako, Gifty Dufie Ampofo

**Affiliations:** 1 Department of Community Health and School of Medicine, University of Health and Allied Sciences, PMB 31, Ho, Volta Region, Ghana; josarfo@uhas.edu.gh (J.O.); ddokyere@gmail.com (D.O.); gampofo@uhas.edu.gh (G.D.A.); 2 Department of Community, College of Medicine and Allied Sciences, North Legon, Accra, Greater Accra Region, Ghana; 3 MARCAD Consortium Secretariat, Faculty of Medicine, University Cheikh Anta Diop of Dakar, Dakar-Fann BP 5005, Senegal; ekoue.kouevidjin@gmail.com; 4 Malaria Centre of the Health Research Institute, University of Health and Allied Sciences, PMB 31, Ho, Volta Region, Ghana; maberese-ako@uhas.edu.gh

**Keywords:** maternal anaemia, low birthweight, malaria in pregnancy, intermittent preventive treatment, iron and folic acid supplementation, antenatal care, household wealth, Ghana

## Abstract

Despite declining malaria transmission, maternal anaemia and low birthweight (LBW) remain high in Ghana, suggesting that non-malarial determinants now dominate these outcomes. A cohort of 5197 pregnant women was enrolled at eight health facilities across Ghana’s Ashanti and Volta regions. Independent predictors of anaemia at term and LBW were identified by multivariable logistic regression using multiple imputation by chained equations. Anaemia at term prevalence was 60.57% and LBW was 11.54%. No intestinal helminths were detected. Anaemia at antenatal care (ANC) booking was the strongest predictor of anaemia at term (aOR 3.98; absolute risk increase of 31.4 percentage points). Three or more iron and folic acid doses and eight or more ANC contacts each reduced risk by only four to six percentage points. For LBW, household poverty (absolute risk increase 8.8%), short maternal stature, and male foetal sex dominated. Intermittent preventive treatment of malaria in pregnancy with sulfadoxine–pyrimethamine (aOR 0.65) remained protective despite booking parasitaemia being non-significant. The Volta region had higher anaemia (71.1%vs. 47.7%) but lower LBW (9.6%vs. 14.4%) than Ashanti, with markedly higher parasite densities among infected women, consistent with waning naturally acquired immunity. Persistent anaemia and LBW reflect nutritional depletion, structural poverty, and increased malaria vulnerability under declining transmission. Pre-conception nutrition, social protection, and sub-microscopic malaria surveillance must complement clinic care.

## 1. Introduction

Maternal anaemia and low birthweight (LBW) are leading contributors to adverse pregnancy outcomes globally, carrying substantial consequences for maternal survival, neonatal health, and long-term child development [1,2,3,4]. Both conditions are inherently multifactorial, arising from complex interactions between infectious diseases, nutritional deficiencies, socioeconomic disadvantage, and the adequacy of healthcare received during pregnancy [5]. Despite decades of programmatic investment, their prevalence remains unacceptably high across sub-Saharan Africa. The 2022 Ghana Demographic and Health Survey (GDHS) reported anaemia in 42–60% of pregnant women and an LBW rate of approximately 10–11% nationally—figures that have changed little over the preceding decade [6].

Among the infectious drivers of these outcomes, malaria in pregnancy has historically occupied a central position. In moderate to high malaria transmission settings, the parasite preferentially sequesters in the placenta, impairing nutrient transfer, triggering inflammatory responses, and causing haemolysis—mechanisms that collectively predispose women to anaemia and restrict foetal growth [7,8,9,10]. In 2023, an estimated 12.4 million pregnancies in moderate- to high-transmission countries were affected by malaria, with West Africa bearing the greatest share: approximately 6 million out of an estimated 16.5 million pregnant women and girls were infected [11]. Seminal work by Brabin and colleagues established that malaria could account for population attributable fractions exceeding 40% for both LBW and maternal anaemia in high-transmission settings [12], providing the epidemiological rationale for the WHO’s recommendations for the intermittent preventive treatment of malaria in pregnancy (IPTp) with sulfadoxine–pyrimethamine (SP) and the widespread scale-up of long-lasting insecticidal nets (LLINs) as the cornerstones of pregnancy protection in malaria-endemic areas [13].

Sustained investment in these interventions has produced measurable gains. Across sub-Saharan Africa, including Ghana, malaria prevalence and parasite densities among pregnant women have declined substantially over the past two decades [14,15]. In Ghana specifically, data from the national District Health Information Management System (DHIMS II) document a consistent downward trend in malaria-related indicators in pregnancy over the past decade [15]. Yet rates of maternal anaemia and LBW have not declined proportionally. A systematic review highlighted this “efficacy–effectiveness gap” more than a decade ago: while clinical trials confirmed that IPTp with SP reduced LBW and maternal anaemia, large-scale programmes in many settings continued to observe persistently high rates of both outcomes [16]. More recent modelling estimates from Walker et al. (2025) suggest that malaria now accounts for a diminishing fraction of the total anaemia burden in many populations where transmission has successfully been reduced [17]. This epidemiological paradox—high burden of maternal anaemia and low birthweight in the context of low transmission—points to a fundamental shift in the relative importance of the factors that drive these outcomes.

As the malaria contribution has declined, non-malarial determinants have risen in relative importance. Nutritional deficiencies—particularly in iron and folate, but also in vitamin B12 and other micronutrients—are increasingly recognised as primary drivers of anaemia and restricted foetal growth, independent of malaria status [18,19]. Cohort studies consistently demonstrate that women who enter pregnancy already nutritionally depleted are at a substantially higher risk of both anaemia and LBW than those with adequate nutritional stores and that antenatal supplementation alone is insufficient to fully reverse this pre-existing deficit [18]. Soil-transmitted helminth infections represent an additional pathway: pooled analyses have reported adjusted odds ratios of 3.09 for hookworm and 1.86 for Ascaris infection in relation to gestational anaemia among women in low- and middle-income countries [20]. Although routine anthelminthic treatment is now a standard component of ANC in Ghana and many other sub-Saharan African countries—a policy designed precisely to mitigate this pathway—the impact of deworming on the residual helminth burden and its downstream consequences for anaemia and birth outcomes warrants direct examination in programmatic settings. Socioeconomic determinants intersect with all of these pathways: poverty restricts dietary diversity and access to care, while lower maternal education and limited antenatal contact are consistently associated with higher risks of preterm delivery, anaemia, and LBW across diverse settings [21].

Ghana provides a particularly valuable setting in which to examine these questions. The country encompasses regions of distinctly different malaria transmission intensity, with historically higher transmission in the forest and coastal zones and lower but persistent transmission in the Volta basin [22]. Ongoing surveillance data suggest that malaria prevalence among pregnant women attending ANC in these regions has declined substantially, yet the burden of anaemia and LBW has not followed [15]. Whether this divergence reflects the growing dominance of nutritional and socioeconomic determinants, the biological consequences of waning naturally acquired immunity, the residual but undetected burden of sub-microscopic malaria, or a combination of all three has not been prospectively characterised in a large, multi-site cohort with detailed clinical, parasitological, and socioeconomic data.

This paper reports the findings of a health-facility-based cohort study of 5197 pregnant women enrolled across the Ashanti and Volta regions of Ghana. The study aimed to determine the prevalence of maternal anaemia at term and LBW and to identify the factors associated with the persistence of high maternal anaemia and low birthweight despite declining malaria transmission and prevalence among a cohort of pregnant women in both regions of Ghana.

## 2. Materials and Methods

### 2.1. Study Design and Setting

This was a health-facility-based non-interventional cohort study of 5197 pregnant women conducted in the Ashanti and Volta Regions of Ghana. The women were enrolled simultaneously at all ANC clinics, in both regions, from May 2018 to March 2020. The study areas were purposively selected to include a representative mix of urban, semi-urban, and rural settings across varying malaria transmission intensities. Detailed descriptions of the study sites, including the specific administrative districts and ecological zones, have been previously published [23].

### 2.2. Sample Size Estimation

The study was originally powered to detect the impact of malaria, a key risk factor for adverse pregnancy outcomes. Assuming a malaria prevalence of 11.0% [24,25], a minimum sample size of 5000 pregnant women and 2500 live births was estimated to provide at least 80% power to detect a population attributable fraction (PAF) of at least 10% for maternal anaemia and low birthweight (LBW), respectively, using methods by Browner and Newman [26].

While malaria was the primary exposure of interest at the design stage, this large sample size also ensured sufficient statistical power for the multivariable logistic regression models used in this study. With a total of 5197 participants and 2921 birthweight records, the study exceeded the requirements for the “rule of thumb” in multivariable modelling (10–15 events per candidate predictor). This allowed for a robust evaluation of diverse non-malarial drivers—including socioeconomic status, maternal anthropometry, and clinical adherence—with high precision and minimal risk of model overfitting.

### 2.3. Participant Enrolment and Procedures

Participant enrolment and procedures have been described in detail previously [23]. In summary, pregnant women were enrolled consecutively during their first ANC visit at eight selected health facilities (hospitals and health centres). Eligibility included women of any age, parity or gestational age willing to comply with ANC schedules during their pregnancy. In Ghana, pregnant women are expected to have eight scheduled ANC contacts as recommended by the WHO.

At enrolment, comprehensive data were collected via electronic structured questionnaires covering socio-demographic and socioeconomic characteristics, obstetric history, and insecticide-treated net (ITN) use. The pregnant women were scheduled for their subsequent visits according to the standard of care by the ANC staff and were followed up passively at these visits. After their routine ANC sessions, the research assistants (RAs) extracted required information from their Maternal and Child Health Record Book (MCHRB). These included presenting complaints, results of physical examinations and laboratory tests, and ANC interventions and treatments received. Blood samples were also collected at enrolment and at each follow-up visit for a full blood count and malaria parasite determination. Urine and stool samples were collected at enrolment for helminth and schistosoma determination. At delivery, blood samples were also collected before the birth of the infant. The number of infants born to a mother, infants’ birthweights and gestational age at delivery were recorded on the day of delivery at the health facilities. If a participant did not deliver at a health facility but reported within 24 h after delivery, study samples were taken and the birthweight was recorded. Any adverse pregnancy outcomes including still births and miscarriages were also recorded. To ensure the target sample size was met, women reporting for delivery who were not previously enrolled were recruited retrospectively using their MCHRB.

### 2.4. Laboratory Investigations

Laboratory investigations conducted in this study have already been reported [23]. Routine ANC data and specific study samples were collected at baseline and delivery. Venous blood was obtained for haemoglobin concentration (Hb), haematocrit and red cell indices using a Sysmex KX-21 analyser and malaria microscopy was conducted to determine malaria parasitaemia. Anaemia was defined as Hb < 11.0 g/dL per the WHO criteria. Stool and urine samples obtained from the women were analysed for helminth and *Schistosoma haematobium* infections using concentration techniques described previously [27].

### 2.5. Nutritional Status Assessment

Maternal pre-pregnancy BMI was derived from height and estimated pre-pregnancy weight, based on the weight recorded at the first ANC visit. Following the methodology validated by Inskip et al. [28] and recently supported in West African settings by Yovo et al. [29], the weight measured before 14 weeks gestation was used as a reliable proxy for pre-pregnancy weight after a standard correction of 0.9 kg was subtracted to account for early gestational gain. For women booking after 14 weeks, pre-pregnancy weight was back-calculated by assuming a first-trimester gain of 0.9 kg and a subsequent weekly gain of 0.4 kg, consistent with IOM guidelines [30,31].

Maternal nutritional status was subsequently categorised based on the calculated pre-pregnancy BMI according to World Health Organization (WHO) standard thresholds [32]. Women were classified as underweight (BMI < 18.5/kg/m^2^), normal weight (BMI = 18.5–24.9/kg/m^2^), overweight (BMI = 25.0–29.9/kg/m^2^), or obese (BMI >30.0/kg/m^2^). Due to the relatively small sample size in the obese category, overweight and obese mothers were collapsed into a single “overweight/obese” group (BMI > 25.0/kg/m^2^) for the multivariable analysis to ensure statistical power and model stability.

### 2.6. Statistical Analysis

Data were collected electronically using personal digital assistants and uploaded to a central database with quality control checks (SurveyCTO). Data were analysed using Stata version 16. Descriptive statistics were used to summarise the baseline characteristics of the study population. Continuous variables are presented as pooled means with 95% confidence intervals (CIs) alongside the observed standard deviations (SDs) from the non-imputed data (m = 0) to describe the sample variance. Categorical variables are reported as frequencies and percentages. Socioeconomic status was determined using a wealth index derived from the principal components analysis (PCA) of housing characteristics and asset ownership [23].

To address missing data and minimise selection bias—particularly given the 50% ANC attrition rate and the 45% non-facility delivery rate identified in national DHIMS data, multiple imputation by chained equations (MICE) was employed. We generated 20 imputed datasets with five iterations each, using a predictive mean matching (PMM) approach for continuous variables to ensure imputed values remained within biologically plausible ranges. All clinical and socioeconomic variables were included in the imputation model. The missing at random (MAR) assumption was justified on the basis that missingness in the key outcomes was primarily driven by identifiable operational and environmental factors (COVID-19 lockdown disruptions and non-facility delivery choices) rather than the unobserved clinical state of the participants. Furthermore, the missingness was modelled conditionally using a comprehensive suite of observed baseline socio-demographic and clinical predictors within the MICE framework.

To evaluate predictors of low birthweight and maternal anaemia at term, multivariable logistic regression models were constructed using both the original (unimputed) and multiple imputed (MI) datasets. Prior to model building, potential covariates were screened for collinearity using Pearson’s correlation (r), where pairs exhibited a correlation coefficient r > 0.5; the most clinically relevant variable was retained to ensure model stability. By this process, gravidity was retained for the “age versus gravidity” pair and wealth quintile retained for the “education versus wealth quintile” pair. Variables demonstrating a univariable association with the outcomes (*p* < 0.1) were then advanced to a backward-fitting multivariable process. During this process, variables were removed in a stepwise, one-at-a-time fashion based on the highest *p*-value, until only those with at least one category demonstrating statistical significance (*p* < 0.05) remained in the final model.

Results are reported as adjusted odds ratios (aORs) with a corresponding 95% CI. For categorical variables with more than two levels, global *p*-values were derived using Wald tests to assess the overall significance of the predictors. Interaction terms between the region of residence and key variables (anaemia at booking, wealth quintile and ANC attendance) were tested to explore the potential effect modification. A sensitivity analysis was performed by comparing the pooled MI estimates with a complete-case analysis (CCA) to evaluate the robustness of the findings. Statistical significance was set at *p* < 0.05.

To enhance the clinical interpretability of the findings, we estimated marginal predicted probabilities (adjusted marginal risks) for the key predictors of maternal anaemia at term and low birthweight. Unlike odds ratios, which represent relative risk, marginal risks provide the absolute predicted probability of the outcomes for each category of a predictor while holding all other covariates in the model at their mean values. These estimates were pooled across the 20 imputed datasets using the **mimrgns** command in Stata. This approach allowed for a direct comparison of the absolute risk increase (ARI) or reduction (ARR) associated with baseline characteristics (nutritional status, anaemia at booking and maternal stature) versus adherence to clinical interventions including IFA supplementation and SP-IPTp uptake across the two study regions.

### 2.7. Ethical Considerations

The study protocol was reviewed and approved by the University of Health and Allied Sciences Research Ethics Committee (Certificate number: **UHAS-REC/A.1 [l] 17-18**). Administrative permissions were further obtained from the relevant district directors of Health Services and the administrative heads of the participating health facilities. All participants provided written informed consent via signature or thumbprint prior to enrolment. To ensure confidentiality, de-identified study identification codes were used for all electronic data capture instead of personal names. The study involved minimal risk to participants, primarily limited to standard venipuncture procedures during routine ANC. All laboratory and data collection procedures were conducted by certified medical laboratory scientists and trained RAs to ensure participant safety and data integrity.

## 3. Results

### 3.1. Baseline Cohort and Participant Flow

**Figure 1:**
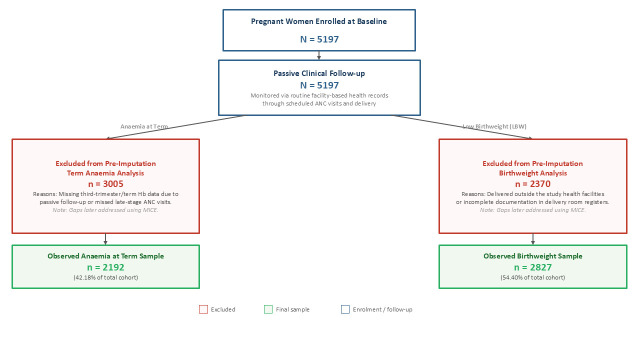
STROBE flow diagram depicting participant progression and analytical sample sizes for maternal anaemia and birthweight outcomes.

A total of 5197 pregnant women were enrolled into the study cohort at baseline across the participating urban, semi-urban, and rural antenatal care (ANC) facilities. Over the course of the passive, facility-based clinical follow-up period, complete baseline socio-demographic and clinical parameters were successfully tracked through routine health records. The progression of participants and the distribution of missing data across the two primary endpoints—maternal anaemia at term and birthweight—are detailed in the STROBE flow diagram (Figure 1).

Due to external operational constraints, including mobility restrictions during the COVID-19 pandemic lockdowns and localised rates of non-facility deliveries, a subset of participants lacked late-stage or delivery-room clinical documentation. Specifically, observed pre-imputation records were available for 2192 women (42.18%) regarding term anaemia status and up to 2921 women (56.21%) regarding complete delivery-room indicators (including newborn sex and birthweights). To maximise statistical power and mitigate the selection bias arising from these missing data structures, multiple imputation by chained equations (MICE) was systematically applied across all missing multivariable model covariates.

### 3.2. Characteristics of the Study Population

The key socio-demographic characteristics of the women are summarised in Table 1. The mean age (SD) of the participants was 27.34 (6.5) years, with 13% (95% CI: 12.11–13.95) being adolescents. Approximately 23% of the women had no formal or only primary education, and 20 to 40% belonged to the poorest wealth quintile. The mean gestational age at booking was 15.5 (8.37) weeks. Fifty percent of the women registered for their first ANC in the first trimester and 23% had the recommended eight or more ANC visits with 37% receiving three or more doses of SP-IPT. Based on their pre-pregnancy BMI, 10% were judged to be underweight and 36.79% overweight or obese. At the time of booking, the mean Hb was 10.69 (1.47) g/dL, which remained relatively stable at 10.62 (1.40) g/dL at term. The pooled prevalence of malaria infection at booking was 6.49% (95% CI, 5.72–7.26) while anaemia (Hb < 11 g/dL) at booking was 55.29% (95% CI, 53.92–56.63). The mean maternal height was 1.59 (0.08) metres.

### 3.3. Prevalence of Low Birthweight and Anaemia at Term

The mean birthweight was 3.06 (0.50) kg, with weights ranging from 1.0 kg to 5.5 kg. The overall pooled prevalence of low birthweight (<2.5 kg) in the study population was 11.54% (95% CI: 10.14–12.93). When stratified by maternal characteristics, the burden of LBW was significantly higher among adolescent women, primigravidae, women who were resident in the Ashanti region, women who had their ANC booking in the third trimester, women who were less than 1.50 m in height, women who were underweight (BMI < 18.5 kg/m^2^) and those who had less than the eight recommended ANC visits, less than the recommended doses of SP-IPTp and iron and folate and were among women of lower quintiles of wealth. (Table 2).

The prevalence of anaemia (Hb < 11 g/dL) at term was 60.57% (95% CI: 58.51–62.63)). When stratified by maternal characteristics, the prevalence of maternal anaemia at term was significantly higher among women who had anaemia at their booking ANC visit after the first trimester, were aged below 20 years, were residents in the Volta region, fell in the lower wealth quintiles, were underweight (BMI <18.5 kg/m^2^), had their ANC booking in the third trimester, had less than the eight recommended ANC visits and had less than the recommended doses of SP-IPTp and iron and folate. (Table 3).

Multivariable predictors and absolute risks for low birthweight (Table 4 and Table 5).

In the unadjusted logistic regression analysis, several factors including the region of residence, maternal age, gravidity, gestation at booking, maternal stature, nutritional status, and number of ANC visits were associated with birthweight (*p* < 0.1).

In the adjusted multivariable analysis, maternal stature, ANC utilization, and socioeconomic status emerged as the strongest independent predictors of neonatal birthweight. After adjusting for confounders, normal maternal stature (≥1.50 m) was associated with 41% lower odds of delivering an LBW infant (aOR: 0.59; 95% CI: 0.43–0.82; *p* = 0.002) compared to shorter women. In absolute terms, this biological advantage translated to the largest clinical impact on LBW observed in the study: an absolute risk reduction (ARR) of 6.13 percentage points (10.51% risk in taller women vs. 16.64% in shorter women).

ANC interventions also showed significant protective effects. Adequate ANC utilization (eight or more visits) was associated with 36% lower odds of LBW (aOR: 0.64; 95% CI: 0.46–0.88; *p* = 0.007), representing an ARR of 3.96 percentage points (8.30% vs. 12.26%). Similarly, although the relative effect for malaria prophylaxis was more modest in the full model, receiving three or more doses of SP-IPTp provided a significant ARR of 4.33 percentage points (9.66% vs. 13.99%; *p* = 0.016).

Finally, a robust socioeconomic gradient persisted; women in the highest wealth quintile had significantly reduced odds of LBW compared to those in the lowest quintile. This was reflected in a substantial 8.79 percentage-point absolute risk increase in LBW for women in the poorest households compared to the wealthiest (16.61% vs. 7.82%, *p* < 0.001).

Multivariable predictors and absolute risks for anaemia at term (Table 6 and Table 7)

In the unadjusted logistic regression analysis, the region of residence, gestational age at booking, maternal stature, nutritional status, anaemia at booking, and the number of antenatal care (ANC) visits and the doses of IFA received were associated with anaemia at term (*p* < 0.1).

In the final multivariable model for maternal anaemia at term, anaemia at booking and region of residence emerged as the most substantial independent predictors. After adjusting for potential confounders, women who presented with anaemia (<11.0 g/dL) at their booking visit had nearly four times the odds of remaining anaemic at term (aOR: 3.98; 95% CI: 3.31–4.79; *p* < 0.001) compared to those with normal haemoglobin. In terms of absolute clinical impact, booking anaemia was associated with a 31.44 percentage-point absolute risk increase (ARI) in term anaemia (74.91% vs. 43.47%, *p* < 0.001). Geography also played a significant role, with residence in the Volta region associated with 2.22 times higher odds of term anaemia (aOR: 2.22; 95% CI: 1.78–2.75; *p* < 0.001) and a predicted probability of 68.17% compared to 51.51% in the Ashanti region.

While clinical interventions were independently associated with improved outcomes, their absolute impact was notably smaller than the influence of ANC booking status. Receiving optimal IFA, three or more doses, significantly reduced the odds of term anaemia (aOR: 0.79; *p* = 0.009), yet this translated to a modest absolute risk reduction (ARR) of only 4.76 percentage points. Similarly, achieving adequate ANC utilization (eight or more visits) provided an ARR of 5.89 percentage points. Consequently, the absolute risk increase driven by starting a pregnancy with a haemoglobin deficit (31.44%) was more than five times the magnitude of the protection offered by completing the full recommended course of ANC visits.

### 3.4. Interaction Analysis for Maternal Anaemia and Low Birthweight

To determine if the observed regional disparities in maternal and neonatal outcomes were modified by clinical, biological, or socioeconomic factors, interaction terms between the region of residence and key predictors were systematically tested for both primary outcomes.

Regarding maternal anaemia at term, no statistically significant interactions were observed between the region of residence and the number of ANC visits (*p* = 0.789), wealth quintiles (*p* = 0.945), or anaemia status at booking (*p* = 0.951). Similarly, the protective effects of optimal IFA supplementation (*p* = 0.312) and maternal nutritional status (*p* = 0.954) did not vary significantly by geographical area.

A similar consistency was observed for neonatal birthweight; no significant interactions were found between the region and maternal stature (*p* = 0.885), wealth quintiles (*p* = 0.786), or the frequency of ANC visits (*p* = 0.404). Furthermore, the influence of infant sex (*p* = 0.352) and maternal BMI (*p* = 0.983) on birthweight remained uniform across both the Ashanti and Volta regions. Collectively, these results indicate that the identified biological, clinical, and socioeconomic determinants of maternal anaemia and low birthweight operate consistently across different geographical contexts, suggesting that the observed regional disparities are likely driven by baseline structural or constitutional differences rather than varying intervention efficacy.

Analysis of the longitudinal progression of maternal anaemia revealed a highly significant interaction between the region of residence and baseline anaemia status at booking (F(1, 47.0) = 203.81, *p* < 0.001). Post-estimation margins indicated that while baseline anaemia increased the risk of term anaemia in both cohorts, the magnitude of this risk was disproportionately higher in the Volta region, where baseline anaemia prevalence was 65.60% compared to 42.6% in the Ashanti region. This suggests that the persistence of anaemia from booking to term is significantly modified by geographical region, characterised by a higher rate of new-onset anaemia or failure of recovery in the Volta population.

Conversely, when evaluating maternal anaemia status specifically at term, no statistically significant interactions were observed between the region of residence and the number of antenatal care (ANC) visits (*p* = 0.789), wealth quintiles (*p* = 0.945), or baseline anaemia status (*p* = 0.951). Furthermore, the protective effects of optimal IFA supplementation (*p* = 0.312) and maternal nutritional status (*p* = 0.954) did not vary significantly by geographical area. These findings indicate that while the baseline burden and persistence of anaemia are regionally distinct, the clinical efficacy of standard ANC interventions and nutritional factors remains uniform across both the Ashanti and Volta regions.

### 3.5. Sensitivity Analysis

To assess the robustness of our findings, we conducted a sensitivity analysis comparing the observed data (complete-case analysis, m = 0) with the pooled results from the multiple imputation (MI) models. Across both outcomes, the direction and magnitude of the effect estimates (aORs) were largely consistent between the observed and imputed datasets (Table 8 and Table 9). For instance, the association between anaemia at booking and term anaemia remained stable in the observed model (aOR: 5.48; 95% CI: [4.20–7.15]) compared to the imputed model (aOR: 3.98; 95% CI: [3.31–4.79]). The use of MI provided higher statistical precision, as evidenced by narrower confidence intervals, but did not alter the fundamental conclusions regarding the key drivers of low birthweight or maternal anaemia at term.

## 4. Discussion

This cohort study of 5197 pregnant women in the Ashanti and Volta regions of Ghana reveals that the persistence of high maternal anaemia and LBW is driven less by ongoing malaria transmission than by three intersecting forces: nutritional depletion that predates pregnancy, structural poverty, and a paradoxical increase in biological vulnerability to malaria in areas where transmission has declined most. These themes are discussed in turn.

The pooled prevalence of anaemia at term (60.57%) and LBW (11.54%) is consistent with national estimates from the 2022 Ghana Demographic and Health Survey (GDHS) and with figures reported from comparable Ghanaian and sub-Saharan African settings [6,33,34]. Ampofo et al. (2025), drawing on baseline data from this same cohort, documented an anaemia prevalence of 55.2% at ANC booking [23], confirming that the deficit was present from the earliest point of contact with the health system. Despite measurable gains in malaria control over the preceding decade, these outcomes—the high prevalence of anaemia at booking ANC and at term pregnancy—remain near the national averages, underscoring the inadequacy of malaria-focused strategies as the sole lever for improving maternal and neonatal health and the improbability of meeting the WHO target of a 30% reduction in LBW by 2025 without broader action [35].

Anaemia at the first ANC visit was by far the strongest independent predictor of anaemia at term (aOR 3.98, 95% CI 3.31–4.79; absolute risk increase of 31.44 percentage points). A woman already anaemic at booking had a predicted probability of approximately 75% of remaining anaemic at delivery, compared with 43% for a non-anaemic counterpart. This confirms that anaemia in pregnancy is largely a continuation of iron and nutritional depletion that predates conception [36,37]—a deficit too large for oral supplementation alone to reverse within nine months, particularly when adherence is incomplete. Receiving three or more IFA doses (aOR 0.79, 95% CI 0.66–0.94; absolute risk reduction of 4.76 percentage points) and completing eight or more ANC contacts (aOR 0.75, 95% CI 0.61–0.91; absolute risk reduction of 5.89 percentage points) were both protective but of modest absolute magnitude [35,38]. These findings argue for a strategic shift towards pre-conception and inter-pregnancy nutrition programmes targeting women of reproductive age, including adolescents—a window of intervention that the current ANC framework cannot reach.

For example, the WHO-recommended intermittent iron and folic acid supplementation is a public health intervention for the purpose of improving pregnancy outcomes and reducing maternal anaemia in pregnancy for adoption by member states [39,40,41]. This includes the Girls’ Iron–Folate Tablet Supplementation (GIFTS) Programme designed to provide adolescent girls with weekly iron and folic acid tablets free of charge to help prevent anaemia in Ghana [42].

Malaria parasitaemia at booking was not independently associated with LBW (*p* = 0.567), yet both adequate ANC attendance (aOR 0.64, 95% CI 0.46–0.88) and three or more doses of SP-IPTp (aOR 0.65, 95% CI 0.46–0.92) remained significantly protective. This apparent inconsistency is explained by the limitations of a single microscopy reading: placental malaria can persist when peripheral blood is clear, and SP-IPTp reduces cumulative parasite burden across pregnancy rather than at any single time point [43,44]. In addition, SP-IPTp could be additionally dealing with sub-microscopic parasites and hence have the positive effect that is being seen. A few studies in Ghana have shown high sub-microscopic prevalence with low microscopic prevalence. Frempong et al., 2023, reported a prevalence of 24%, against 8% by microscopy, in pregnant women booking ANC in the Central Tongu district of Volta region [45] and Quakyi et al., 2019, reported an average prevalence of 43% using ultrasensitive PCR techniques against an average prevalence of 4% using microscopy among pregnant women booking ANC in the Greater Accra region [46]. Similarly, a prevalence of 44% in the dry season and 47% in the rainy season has been reported by Anabire et al., 2023, in the Central region using PCR techniques [47].

Maternal short statures below 150 cm (aOR 0.59, 95% CI 0.43–0.82; absolute risk reduction of 6.13%) and belonging to the poorest household wealth quintile (aOR 0.41, 95% CI 0.26–0.66 for richest versus poorest; absolute risk increase of 8.8%) emerged as the dominant determinants of LBW [48,49]. Both reflect a lifelong and intergenerational nutritional disadvantage that cannot be addressed within any single pregnancy. Male foetal sex was also independently associated with higher LBW risk (aOR 1.37, 95% CI 1.10–1.69), consistent with the greater metabolic vulnerability of male foetuses to nutrient restriction [50]. Collectively, the absolute risk differences attributable to structural factors substantially exceeded those achievable through clinical interventions, which reduced risk by only four to six percentage points even under optimal conditions.

No intestinal helminth infections were detected in adequately examined stool samples. This is consistent with the low prevalence of soil-transmitted helminths found by a few studies among pregnant women in Ghana between 2012 and 2018 [51,52,53,54]. Most plausibly, this downward trend reflects the protective effect of routine anthelmintic treatment administered as part of the standard ANC protocol, in line with WHO preventive chemotherapy guidelines [55]. From a diagnostic standpoint, the use of the formalin ethyl-acetate concentration technique minimises the risk of false negatives, as it is highly effective at the recovery of ova and larvae even in low-intensity infections. Therefore, this absolute absence of detectable parasites provides robust evidence that periodic chemotherapy, potentially reinforced by regional improvements in water, sanitation, and hygiene (WASH) infrastructure [56,57], is successfully suppressing transmission cycles within this cohort. While single-sample microscopic protocols can still miss ultra-low biomass infections, a zero-prevalence result via concentration methodologies is a significant epidemiological milestone, signalling a shift from infection control toward localised elimination. While this precludes quantifying a helminth contribution to anaemia in this cohort, the finding affirms that routine deworming is functioning as intended and should be sustained as a vital component of maternal health policy [58].

The combined absolute risk reduction achievable through full adherence to the evaluated clinical ANC components was approximately four to six percentage points for both outcomes—substantially less than the risk attributable to poverty and pre-existing nutritional status. This ceiling effect is consistent with evidence that the largest remaining gains in maternal and child health in low- and middle-income countries will come from upstream interventions addressing food security, income inequality, and women’s education rather than from further refinements to clinical protocols [59,60]. Achieving the WHO LBW target will require social protection policies—including food supplementation, conditional cash transfers, and community-based nutrition support—deployed in parallel with, not instead of, clinical care.

The most novel finding of this study is the striking divergence between the two regions. Anaemia at term was substantially higher in the Volta region (71.05%) than in Ashanti (47.69%), yet the predicted risk of LBW was lower in the Volta region (9.6% versus 14.4%). The key to this paradox may lie in parasite density: among women with confirmed infection at booking, the geometric mean parasite density was 18,226 parasites per microlitre in the Volta region versus only 982 per microlitre in the Ashanti region—approximately 18 times higher—despite greater [67% (95% CI: 65.30–68.96)] ITN use and lower [2.69% (95% CI: 2.1–3.3)] malaria prevalence in Volta.

This pattern is consistent with the recognised consequence of sustained vector control: as transmission declines and repeat exposure to parasites becomes less frequent, the naturally acquired immunity accumulated over years of high-transmission exposure begins to wane [61,62]. Pregnant women in the Volta region, already immunologically more vulnerable during pregnancy [63,64], enter gestation with a lower baseline level of protective immunity than their counterparts in higher-transmission Ashanti. When infection does occur, the immune response is less able to contain parasite replication, resulting in higher parasite densities despite low prevalence. Conventional light microscopy also has a detection threshold of 50–200 parasites per microlitre hence sub-microscopic infections, which are more prevalent in low-transmission settings, remain invisible to routine diagnosis and are independently associated with maternal anaemia and placental inflammation [65,66]. The higher anaemia burden in Volta likely reflects this combination of eroded immunity and undetected sub-microscopic parasitaemia. The relatively better birthweight outcomes in the same region may reflect the fact that nutritional anaemia, however severe, does not impair placental function in the same direct structural manner as active placental malaria infection [44,64], which may be proportionally less common in Volta despite its higher parasite densities.

The policy implication is important: declining malaria prevalence and increasing ITN coverage—the standard metrics of programme success—are insufficient proxies for declining harm to pregnant women. In populations where transmission has fallen rapidly, the erosion of naturally acquired immunity means that residual infections may cause disproportionate harm. Programme monitoring should incorporate parasite density alongside prevalence and molecular diagnostics capable of detecting sub-microscopic infections should be considered for sentinel surveillance in low-transmission settings. The continued relevance of IPTp in areas approaching very low transmission warrants active re-evaluation.

**Strengths and limitations**

This study has several important methodological strengths. The large, geographically diverse cohort of 5197 pregnant women, enrolled consecutively across urban, semi-urban, and rural health facilities in two ecologically and epidemiologically distinct regions, provides a broadly representative sample of women accessing antenatal care in Ghana. The use of multiple imputation by chained equations (MICE) across 20 imputed datasets to handle missing data—particularly for birthweight (45.6% missing) and anaemia at term (57.8% missing), both driven primarily by the abrupt stop of follow-up due to the lockdown of COVID 19 and high rate of non-facility delivery—reduces the potential for selection bias that would affect a complete-case analysis. This approach relies on the missing at random (MAR) assumption. In this cohort, MAR is strongly justified because missingness was predominantly triggered by exogenous, non-clinical disruptions—specifically, the strict mobility restrictions imposed during the COVID-19 lockdown and established regional rates of non-facility deliveries—rather than the unobserved health conditions of the patients. By including a wide array of completely observed baseline variables (such as wealth quintiles, education, and booking clinical status) in the imputation models, we captured the systematic variance associated with missingness. The consistency between the multiple imputation and complete-case analyses for the principal predictors of both outcomes strengthens confidence in the robustness of the findings. The estimation of adjusted marginal risks alongside odds ratios enables direct comparison of the absolute magnitude of effects attributable to clinical interventions versus structural determinants—a distinction that is often obscured when only relative risk measures are reported.

Several limitations should be acknowledged. First, the study was conducted entirely among women who attended health facilities for ANC; women who did not access formal ANC, who delivered outside the health system, or who were lost to follow-up are not represented. These women may be disproportionately among the most socioeconomically disadvantaged, with potentially worse outcomes, meaning that the true population-level burden of anaemia and LBW may be higher than these estimates suggest. Second, the reliance on conventional light microscopy for malaria diagnosis means that sub-microscopic infections—which are particularly prevalent in low-transmission settings—would have been systematically missed, potentially underestimating the contribution of malaria to both anaemia and LBW, especially in the Volta region. Third, dietary intake and food security were not directly assessed in this study; household wealth and pre-pregnancy BMI were used as proxies for nutritional status, which, while validated in similar settings, did not capture the full complexity of individual dietary exposures. Finally, as an observational cohort study, the design does not permit causal inference: the associations reported here should be interpreted as independent predictors, not proven causes, and residual confounding by unmeasured factors cannot be excluded.

## 5. Conclusions

This study demonstrates that the persistence of high maternal anaemia and LBW in Ghana is not primarily driven by ongoing malaria transmission. Rather, it reflects the combined influence of pre-existing nutritional depletion that antedates pregnancy, the deep structural disadvantages associated with household poverty, and a changing malaria epidemiological landscape in which declining transmission has paradoxically increased the biological vulnerability of pregnant women in lower-transmission areas. Clinical interventions—including SP-IPTp, iron and folate supplementation, and structured ANC attendance—remain essential components of pregnancy care and should be sustained and improved. However, their capacity to reduce the absolute burden of anaemia and LBW is inherently limited. Achieving the WHO target of a 30% reduction in LBW will require a broader response that combines clinical care with pre-conception nutrition programmes, social protection policies that address household food security and poverty, and diagnostic systems capable of detecting sub-microscopic malaria infections that conventional microscopies miss. The regional paradox described in this study—declining malaria prevalence accompanied by increasing biological susceptibility and divergent health outcomes—should caution policymakers and programme managers against equating progress in malaria control with equivalent improvements in pregnancy outcomes. Declining transmission may introduce new forms of vulnerability that require active and targeted surveillance, re-evaluation of the IPTp policy in low-transmission settings, and heightened attention to the nutritional and socioeconomic foundations of maternal health.

## Figures and Tables

**Table 1:** Study of women’s characteristics: A pooled multiple imputation analysis.

Variables	Number of Participants	% (95% CI)
**Region of residence**		
Ashanti region	2333	44.89 (43.54–46.24)
Volta region	2864	55.11 (53.76–56.46)
**Maternal Age (years)**		
<20 yrs	670	13.01 (12.1–13.93)
20–34 yrs	3679	71.42 (70.18–72.65)
>=35 yrs	804	15.57 (14.58–16.56)
**Gravidity**		
Primigravidae	1188	23.09 (21.94–24.24)
Secundigravidae	1131	21.97 (20.84–23.1)
Multigravidae	2822	54.94 (53.58–56.3)
**Gestational age at booking**		
1st Trimester (<14 wks)	2478	49.96 (48.57–51.35)
2nd Trimester (14–27 wks)	1981	40.72 (39.34–42.1)
3rd Trimester (28+ wks)	466	9.32 (8.52–10.12)
**Level of education attained**		
Up to primary education	1203	23.42 (22.26–24.58)
Secondary education	3460	67.28 (66–68.57)
Tertiary education	478	9.3 (8.51–10.1)
**Wealth Quintiles (20% each)**		
Lower	1029	20.01 (18.92–21.09)
Lower middle	1028	20 (18.91–21.09)
Middle	1028	20.01 (18.92–21.09)
Upper middle	1028	20 (18.91–21.09)
Upper	1028	19.99 (18.9–21.07)
**Maternal Height < 150 cm**		
Short (<150 cm)	335	11.96 (10.77–13.14)
Normal (>=150 cm)	3211	88.04 (86.86–89.23)
**Pre-pregnancy BMI**		
Underweight (<18.5)	335	11.07 (10–12.13)
Normal (18.5–24.9)	1730	48.8 (47.15–50.45)
Overweight/Obese (>=25.0)	1202	40.14 (38.41–41.86)
**Sex of newborn**		
Female	1601	55.34 (53.09–57.6)
Male	1320	44.66 (42.4–46.91)
**Number of ANC visits**		
Inadequate <8	4015	77.26 (76.12–78.4)
Adequate =/>8	1182	22.74 (21.6–23.88)
**Number of SP-IPTp doses**		
None	1307	25.34 (24.15–26.53)
Up to 2 doses	1924	37.3 (35.98–38.62)
3 or more doses	1927	37.36 (36.04–38.68)
**Received >=iron and folate doses**		
No	2303	45.73 (44.35–47.11)
Yes	2733	54.27 (52.89–55.65)
**Malaria parasitaemia at booking**		
No	4432	93.51 (92.74–94.27)
Yes	270	6.49 (5.73–7.26)
**Hb status at booking (g/dL)**		
Severe anaemia (Hb < 7)	77	1.49 (1.16–1.82)
Moderate anaemia (Hb = 7.0–9.9)	1344	26.07 (24.87–27.27)
Mild Anaemia (Hb = 10.0–10.9)	1426	27.66 (26.44–28.88)
No Anaemia (Hb ≥ 11.0)	2309	44.78 (43.43–46.14)
**Symptoms reported at booking**		
No symptoms	2813	54.6 (53.24–55.96)
Has only one symptom	1078	20.9 (19.79–22.01)
Has two or more symptoms	1264	24.5 (23.32–25.67)
**High systolic/diastolic BP at booking**		
No	3634	91.53 (90.61–92.44)
Yes	352	8.47 (7.56–9.39)

ANC = antenatal clinic; Hb = haemoglobin concentration; BP = blood pressure; SP-IPTp = intermittent preventive treatment of malaria in pregnancy with sulfadoxine–pyrimethamine; BMI = body mass index; reported counts (n) represent the observed data (m = 0). Percentages and confidence intervals are pooled estimates derived from 20 imputed datasets using multiple imputation by chained equations (MICE) to account for missingness.

**Table 2:** Prevalence and predictors of low birthweight (<2.5 kg) among the studied women.

Low Birthweight (<2.5 kg)				
	Total Number of Women (N)	Yes (n)	% (95% CI)	Pooled *p*-Value
**Region of residence**				
Ashanti region	1446	141	12.9 (10.77–15.02)	0.0606
Volta region	1381	102	10.43 (8.72–12.13)	
**Maternal Age (years)**				
<20	369	49	17.43 (12.65–22.22)	0.0014
20–34	1987	157	10.86 (9.43–12.28)	
≥35	452	34	9.71 (7.08–12.35)	
**Gravidity**				
Primigravidae	645	74	15.97 (12.8–19.15)	0.0001
Secundigravidae	617	57	11.96 (9.27–14.65)	
Multigravidae	1541	109	9.5 (8.1–10.9)	
**Gestational age at booking (weeks)**				
1st Trimester (<14)	1245	86	10.12 (8.34–11.89)	0.0009
2nd Trimester (14–27)	1144	92	11.96 (10.12–13.8)	
3rd Trimester (≥28)	334	55	17.28 (13.29–21.27)	
**Level of education attained**				
Up to primary education	593	64	13.31 (10.64–15.98)	0.1436
Secondary education	1939	163	11.32 (9.51–13.12)	
Tertiary education	271	13	8.66 (5.29–12.02)	
**Wealth Quintiles**				
Lower	484	62	15.86 (12.93–18.78)	0.0003
Lower middle	519	46	12.76 (9.98–15.53)	
Middle	557	44	10.72 (8.05–13.4)	
Upper middle	552	46	10.3 (7.82–12.79)	
Upper	691	42	8.03 (5.84–10.23)	
**Maternal Height (cm)**				
Normal (≥15)	1428	113	10.67 (9.24–12.09)	0.0001
Short (<150)	145	19	17.95 (13.87–22.04)	
**Pre-pregnancy BMI**				
Underweight (<18.5)	142	11	15.79 (11.96–19.62)	0.0001
Normal (18.5–24.9)	758	74	13.03 (11.14–14.92)	
Overweight/Obese (≥25.0)	537	38	8.56 (6.77–10.34)	
**Sex of newborn**				
Female	1540	117	10.23 (8.58–11.89)	0.0075
Male	1284	125	13.14 (11.28–15.01)	
**Number of ANC visits**				
Inadequate <8	1800	181	13.04 (11.29–14.79)	<0.001
Adequate ≥8	1027	62	6.44 (4.93–7.95)	
**Number of SP-IPTp doses**				
None	347	41	15.05 (11.76–18.35)	0.0004
Up to 2 doses	1000	96	12.23 (9.97–14.49)	
3 or more doses	1465	104	8.40 (6.85–9.94)	
**Received **≥**3IFA doses**				
No	846	88	14.27 (11.92–16.61)	0.0001
Yes	1844	130	8.87 (7.34–10.4)	
**Slept under ITN night prior to enrolment**				
No	907	82	10.99 (8.74–13.23)	0.0001
Yes	1125	90	11.56 (9.77–13.34)	
**Malaria parasitaemia at booking**				
No	2233	205	11.42 (10–12.85)	0.567
Yes	154	13	13.18 (7.2–19.16)	
**Hb status at booking (g/dL)**				
Severe anaemia (Hb < 7)	42	6	16 ( 6–27)	<0.001
Moderate anaemia (Hb = 7.0–9.9)	701	69	13 (10–15)	
Mild anaemia (Hb = 10.0–10.9)	801	73	12 (9–14)	
No anaemia (Hb ≥ 11.0)	1280	95	11 (9–13)	
**High systolic/diastolic BP at booking**				
No	1598	134	11.46 (9.96–12.96)	0.701
Yes	165	18	12.33 (8.30–16.37)	

ITN = insecticide-treated nets; IFA = iron and folic acid; reported counts (n) and denominators (N) represent the observed data (m = 0). Percentages, confidence intervals and *p*-values are pooled estimates derived from 20 imputed datasets using multiple imputation by chained equations (MICE) to account for missingness.

**Table 3:** Prevalence and predictors of anaemia at term among the studied women.

Maternal Anaemia at Term (<11 g/dL)				
	Total Number of Women (N)	Yes (n)	% (95% CI)	Pooled *p*-Value
**Region of residence**				
Ashanti region	1036	481	47.69 (44.76–50.63)	<0.001
Volta region	1156	800	71.05 (68.38–73.74)	
**Maternal Age (years)**				
<20	290	197	70.11 (65.63–74.6)	<0.001
20–34	1540	887	59.56 (57.28–61.84)	
≥35	356	193	57.21 (53.03–61.39)	
**Gravidity**				
Primigravidae	487	297	62.45 (58.47–66.44)	0.3347
Secundigravidae	492	289	61.4 (57.95–64.86)	
Multigravidae	1201	688	59.44 (56.78–62.1)	
**Gestational age at booking (weeks )**				
1st Trimester (<14)	996	534	56.62 (53.78–59.46)	0.0002
2nd Trimester (14–27)	974	588	64.37 (61.82–66.92)	
3rd Trimester (≥28)	132	92	65.1 (58.3–71.92)	
**Level of education attained**				
Up to primary education	501	330	67.78 (64.25–71.33)	<0.001
Secondary education	1484	859	59.73 (57.33–62.14)	
Tertiary education	195	85	48.43 (41.71–55.16)	
**Wealth Quintiles**				
Lower	429	301	70.79 (66.87–74.73)	<0.001
Lower middle	387	257	67.09 (62.94–71.26)	
Middle	367	218	61.69 (57.46–65.92)	
Upper middle	416	220	54.63 (50.65–58.61)	
Upper	581	278	48.61 (44.93–52.3)	
**Maternal Height (cm)**				
Short (<150)	131	67	54.13 (48.86–59.41)	0.0061
Normal (≥150)	1168	728	61.39 (59.36–63.43)	
**Pre-pregnancy BMI**				
Underweight (<18.5)	132	102	72.81 (67.66–77.98)	<0.001
Normal (18.5–24.9)	648	410	64.45 (61.44–67.48)	
Overweight/Obese (≥25.0)	453	236	52.47 (49.23–55.72)	
**Sex of newborn**				
Female	1163	684	60.98 (58.26–63.71)	0.5886
Male	1015	588	60.05 (57.47–62.63)	
**Number of ANC visits**				
Inadequate <8	1435	898	63.15 (60.68–65.62)	<0.001
Adequate ≥8	757	383	51.79 (48.34–55.25)	
**Number of SP-IPTp doses**				
None	273	163	59.65 (55.36–63.94)	0.0082
Up to 2 doses	760	470	64.62 (60.95–68.31)	
3 or more doses	1154	645	57.06 (54.14–59.98)	
**Received **≥**3 IFA doses**				
No	656	451	64.32 (61.3–67.35)	0.0004
Yes	1514	817	57.53 (55.15–59.92)	
**Slept under ITN night ** **prior to enrolment**				
No	636	347	57.85 (54.58–61.10)	<0.001
Yes	917	597	65.99 (62.96–69.02)	
**Malaria parasitaemia ** **at booking**				
No	1726	1010	60.28 (58.16–62.41)	0.3008
Yes	117	74	64.64 (56.63–72.66)	
**Hb status at booking (g/dL)**				
Severe anaemia (Hb < 7)	37	32	93 (87–99)	<0.001
Moderate anaemia (Hb = 7.0–9.9)	567	478	85 (82–87)	
Mild Anaemia (Hb = 10.0–10.9)	616	429	68 (65–72)	
No Anaemia (Hb ≥ 11.0)	969	339	41 (38–43)	
**High systolic/diastolic BP at booking**				
No	1260	769	60.66 (58.65–62.68)	0.7749
Yes	146	88	59.54 (51.9–67.19)	

Reported counts (n) and denominators (N) represent the observed data (m = 0). Percentages, confidence intervals and *p*-values are pooled estimates derived from 20 imputed datasets using multiple imputation by chained equations (MICE) to account for missingness.

**Table 4:** Unadjusted and adjusted odds ratios of factors associated with low birthweight among pregnant women in Ghana.

	Low Birthweight (<2.5 kg)					
	Total Number of Women (N)	Yes (n)	Unadjusted OR (95% CI)	*p*-Value	Adjusted OR (95% CI)	*p*-Value
**Region of residence**						
Ashanti region	1446	141	1.00 (Ref)	.	1.00 (Ref)	.
Volta region	1381	102	0.79 (0.61–1.01)	0.061	0.62 (0.45–0.85)	0.003
**Gravidity**						
Primigravidae	645	74	1.00 (Ref)	.	1.00 (Ref)	.
Secundigravidae	617	57	0.71 (0.51–1.00)	0.05	0.79 (0.55–1.13)	0.192
Multigravidae	1541	109	0.55 (0.43–0.72)	<0.001	0.61 (0.45–0.82)	0.001
**Gestational age at booking (weeks)**						
1st Trimester (<14)	1245	86	1.00 (Ref)	.	—	—
2nd Trimester (14–27)	1144	92	1.21 (0.97–1.51)	0.093	—	—
3rd Trimester (≥28)	334	55	1.86 (1.35–2.56)	<0.001	—	—
**Wealth Quintiles**						
Lower	484	62	1.00 (Ref)	.	1.00 (Ref)	.
Lower middle	519	46	0.78 (0.57–1.06)	0.105	0.69 (0.50–0.96)	0.028
Middle	557	44	0.64 (0.46–0.87)	0.005	0.60 (0.43–0.82)	0.002
Upper middle	552	46	0.61 (0.44–0.84)	0.002	0.57 (0.39–0.82)	0.003
Upper	691	42	0.46 (0.32–0.67)	<0.001	0.41 (0.26–0.66)	<0.001
**Maternal Height (cm)**						
Short (<150)	145	19	1.00 (Ref)	.	1.00 (Ref)	.
Normal (≥150)	1428	113	0.55 (0.41–0.73)	<0.001	0.59 (0.43–0.82)	0.002
**Pre-pregnancy BMI (kg/m^2^)**						
Underweight (<18.5)	142	11	1.00 (Ref)	.	1.00 (Ref)	.
Normal (18.5–24.9)	758	74	0.80 (0.58–1.10)	0.163	0.87 (0.62–1.22)	0.414
Overweight/Obese (≥25.0)	537	38	0.50 (0.35–0.71)	<0.001	0.63 (0.42–0.94)	0.025
**Sex of newborn**						
Female	1540	117	1.00 (Ref)	.	1.00 (Ref)	.
Male	1284	125	1.33 (1.08–1.63)	0.007	1.37 (1.10–1.69)	0.004
**Number of ANC visits**						
Inadequate <8	1800	181	1.00 (Ref)	.	1.00 (Ref)	.
Adequate ≥8	1027	62	0.46 (0.34–0.62)	<0.001	0.64 (0.46–0.88)	0.007
**Number of SP-IPTp doses**						
None	347	41	1.00 (Ref)	.	1.00 (Ref)	.
Up to 2 doses	1000	96	0.79 (0.57–1.08)	0.139	0.79 (0.57–1.09)	0.145
3 or more doses	1465	104	0.52 (0.38–0.71)	<0.001	0.65 (0.46–0.92)	0.016
**Optimum ANC package received**						
No	317	49	1.00 (Ref)	.	—	—
Yes	2501	193	0.57 (0.43–0.75)	<0.001	—	—
**Received ≥3 iron and folate doses**						
No	846	88	1.00 (Ref)	.	—	—
Yes	1844	130	0.58 (0.45–0.76)	<0.001	—	—
**Slept under ITN night prior to enrolment**						
No	907	82	1.00 (Ref)		—	—
Yes	1125	90	1.06 (0.80–1.40)	0.678	—	—
**Malaria parasitaemia at booking**						
No	2233	205	1.00 (Ref)	.	—	—
Yes	154	13	1.16 (0.69–1.97)	0.567	—	—
**Anaemia Status at Booking (<11 g/dL)**						
No	1280	95	1.00 (Ref)	.	—	—
Yes	1544	148	1.17 (0.93–1.48)	0.177	—	—
**High systolic/diastolic BP at booking**						
No	1598	134	1.00 (Ref)	.	—	—
Yes	165	18	1.08 (0.72–1.63)	0.701	—	—

OR = odds ratio; Note: Reported counts (n) and denominators (N) represent the observed data (m = 0). Odds ratios, confidence intervals and *p*-values are pooled estimates derived from 20 imputed datasets using multiple imputation by chained equations (MICE) to account for missingness. Adjusted Odds Ratios: Model adjusted for all variables listed in the table.

**Table 5:** Multivariable associations and adjusted marginal risks for low birthweight (N = 5197).

Predictor Variable	Adjusted Marginal Risk (%) (95% CI)	Absolute Risk Difference	Adjusted OR (95% CI)	*p*-Value
**Region of residence**				
Ashanti region	14.37 (11.78–16.95)			
Volta region	9.56 (7.93–11.2)	4.81	0.62 (0.45–0.85)	0.003
**Number of ANC visits**				
Inadequate <8	12.26 (10.64–13.89)			
Adequate ≥8	8.3 (6.17–10.42)	3.96	0.64 (0.46–0.88)	0.007
**Number of SP-IPTp doses**				
None	13.99 (10.95–17.03)			
Up to 2 doses	11.41 (9.25–13.56)		0.79 (0.57–1.09)	0.145
3 or more doses	9.66 (7.7–11.61)	4.33	0.65 (0.46–0.92)	0.016
**Maternal Height (cm)**				
Normal (≥150)	16.64 (12.67–20.61)			
Short (<150)	10.77 (9.34–12.2)	5.87	0.59 (0.43–0.82)	0.002
**Sex of newborn**				
Female	10.15 (8.51–11.79)			
Male	13.23 (11.39–15.06)	3.08	1.37 (1.1–1.69)	0.004
**Pre-pregnancy BMI (kg/m^2^)**				
Underweight (<18.5)	14.04 (10.42–17.67)			
Normal (18.5–24.9)	12.46 (10.62–14.29)		0.87 (0.62–1.22)	0.414
Overweight/Obese (≥25.0)	9.42 (7.35–11.48)	4.62	0.63 (0.42–0.94)	0.025
**Wealth Quintiles**				
Lower	16.61 (13.24–19.97)			
Lower middle	12.27 (9.58–14.95)		0.69 (0.5–0.96)	0.028
Middle	10.79 (8.18–13.41)		0.6 (0.43–0.82)	0.002
Upper middle	10.35 (7.82–12.89)		0.57 (0.39–0.82)	0.003
Upper	7.82 (5.4–10.24)	8.79	0.41 (0.26–0.66)	<0.001
**Gravidity**				
Primigravidae	14.86 (11.71–18.01)			
Secundigravidae	12.2 (9.46–14.94)		0.79 (0.55–1.13)	0.192
Multigravidae	9.73 (8.23–11.23)	5.13	0.61 (0.45–0.82)	0.001

N = 5197: Total study population; estimates are pooled across 20 imputed datasets using multiple imputation by chained equations (MICE) to account for missing values in predictors and outcomes. aOR (Adjusted Odds Ratio): Derived from multivariable logistic regression models (Table 4 and Table 6). Adjusted Marginal Risk (%): Represents the predicted absolute probability of the outcome for a given category, holding all other covariates in the model at their observed means (the “average” participant). Ref. (Reference): The baseline category against which the odds ratio and risk difference are compared. *p*-values are derived from the multivariable logistic regression model (aOR).

**Table 6:** Unadjusted and adjusted odds ratios of factors associated with maternal anaemia at term among pregnant women in Ghana.

	Maternal Anaemia at Term (<11 g/dL)					
	Total Number of Women (N)	Yes (n)	Unadjusted OR (95% CI)	*p*-Value	Adjusted OR (95% CI)	*p*-Value
**Region of residence**						
Ashanti region	1036	481	1.00 (Ref)	.	1.00 (Ref)	.
Volta region	1156	800	2.69 (2.27–3.19)	<0.001	2.22 (1.78–2.75)	<0.001
**Gravidity**						
Primigravidae	487	297	1.00 (Ref)	.	—	—
Secundigravidae	492	289	0.96 (0.77–1.19)	0.691	—	—
Multigravidae	1201	688	0.88 (0.74–1.05)	0.159	—	—
**Gestational age at booking (weeks)**						
1st Trimester (<14)	996	534	1.00 (Ref)	.	1.00 (Ref)	.
2nd Trimester (14–27)	974	588	1.38 (1.20–1.59)	<0.001	1.12 (0.93–1.34)	0.229
3rd Trimester (≥28)	132	92	1.43 (1.03–1.99)	0.032	1.17 (0.78–1.75)	0.45
**Wealth Quintiles**						
Lower	429	301	1.00 (Ref)	.	—	—
Lower middle	387	257	0.84 (0.64–1.10)	0.203	—	—
Middle	367	218	0.66 (0.51–0.86)	0.002	—	—
Upper middle	416	220	0.50 (0.40–0.62)	<0.001	—	—
Upper	581	278	0.39 (0.31–0.49)	<0.001	—	—
**Maternal Height (cm)**						
Normal (≥150)	131	67	1.00 (Ref)	.	—	—
Short (<150)	1168	728	1.35 (1.09–1.66)	0.006	—	—
**Pre** **-pregnancy BMI (kg/m^2^)**						
Underweight (<18.5)	132	102	1.00 (Ref)	.	1.00 (Ref)	.
Normal (18.5–24.9)	648	410	0.68 (0.51–0.89)	0.006	0.91 (0.66–1.26)	0.575
Overweight/Obese (≥25.0)	453	236	0.41 (0.30–0.56)	<0.001	0.86 (0.58–1.27)	0.438
**Sex of newborn**						
Female	1163	684	1.00 (Ref)	.	—	—
Male	1015	588	0.96 (0.83–1.11)	0.589	—	—
**Number of ANC visits**						
Inadequate <8	1435	898	1.00 (Ref)	.	1.00 (Ref)	.
Adequate ≥8	757	383	0.63 (0.53–0.75)	<0.001	0.77 (0.63–0.94)	0.013
**Number of SP-IPTp doses**						
None	273	163	1.00 (Ref)	.	—	—
Up to 2 doses	760	470	1.24 (1.00–1.53)	0.049	—	—
3 or more doses	1154	645	0.90 (0.71–1.14)	0.364	—	—
**Received ≥3 IFA doses**						
No	656	451	1.00 (Ref)	.	1.00 (Ref)	.
Yes	1514	817	0.75 (0.64–0.88)	<0.001	0.80 (0.66–0.96)	0.015
**Slept under ITN night prior to enrolment**						
No	636	347	1.00 (Ref)		—	—
Yes	917	597	1.41 (1.2–1.67)	<0.001	—	—
**Malaria parasitaemia at booking**						
No	1726	1010	1.00 (Ref)	.	—	—
Yes	117	74	1.21 (0.84–1.73)	0.301	—	—
**Anaemia Status at Booking (<11 g/dL)**						
No	969	339	1.00 (Ref)	.	1.00 (Ref)	.
Yes	1220	939	4.81 (4.05–5.70)	<0.001	3.98 (3.31–4.79)	<0.001
**High systolic/diastolic BP at booking**						
No	1260	769	1.00 (Ref)	.	—	—
Yes	146	88	0.96 (0.70–1.31)	0.775	—	—

Reported counts (n) and denominators (N) represent the observed data (m = 0). Odds ratios, confidence intervals and *p*-values are pooled estimates derived from 20 imputed datasets using multiple imputation by chained equations (MICE) to account for missingness. Adjusted Odds Ratios: Model adjusted for all variables listed in the table.

**Table 7:** Multivariable associations and adjusted marginal risks for maternal anaemia at term (n = 5197).

Predictor Variable	Adjusted Marginal Risk (%) (95% CI)	Absolute Risk Difference	Adjusted OR (95% CI)	*p*-Value
**Region of residence**				
Ashanti region	51.51 (48.69–54.34)			
Volta region	68.17 (65.46–70.88)	−16.66	2.22 (1.84–2.68)	< 0.001
**Anaemia Status at Booking (<11 g/dL)**				
No	43.47 (40.68–46.26)			
Yes	74.91 (72.33–77.49)	−31.44	4.13 (3.47–4.92)	< 0.001
**Received ≥ 3 iron and folate doses**				
No	63.24 (60.35–66.13)			
Yes	58.48 (56.11–60.84)	4.76	0.79 (0.66–0.94)	0.009
**Number of ANC visits**				
Inadequate <8	62.01 (59.71–64.32)			
Adequate ≥8	56.12 (52.85–59.4)	5.89	0.75 (0.61–0.91)	0.003

N = 5197: Total study population; estimates are pooled across 20 imputed datasets using multiple imputation by chained equations (MICE) to account for missing values in predictors and outcomes. aOR (Adjusted Odds Ratio): Derived from multivariable logistic regression models (Table 4 and Table 6). Adjusted Marginal Risk (%): Represents the predicted absolute probability of the outcome for a given category, holding all other covariates in the model at their observed means (the “average” participant). Ref. (Reference): The baseline category against which the odds ratio and risk difference are compared. *p*-values are derived from the multivariable logistic regression model (aOR).

**Table 8:** Comparison of factors associated with low birthweight using multiple imputation and complete-case analysis.

	Multiple Imputation (Pooled)	Complete-Case Analysis
	N = 5054	N = 1396
	Adjusted OR (95% CI)	Adjusted OR (95% CI)
**Region of residence**		
Ashanti region	1.00 (Ref)	1.00 (Ref)
Volta region	0.62 (0.45–0.85)	0.91 (0.58–1.44)
**Gravidity**		
Primigravidae	1.00 (Ref)	1.00 (Ref)
Secundigravidae	0.79 (0.55–1.13)	1.00 (0.57–1.76)
Multigravidae	0.61 (0.45–0.82)	0.85 (0.53–1.38)
**Wealth Quintiles**		
Lower	1.00 (Ref)	1.00 (Ref)
Lower middle	0.69 (0.50–0.96)	0.63 (0.37–1.09)
Middle	0.60 (0.43–0.82)	0.55 (0.31–1.00)
Upper middle	0.57 (0.39–0.82)	0.85 (0.48–1.50)
Upper	0.41 (0.26–0.66)	0.43 (0.20–0.91)
**Maternal Height (cm)**		
Normal (≥150)	1.00 (Ref)	1.00 (Ref)
Short (<150)	0.59 (0.43–0.82)	0.62 (0.35–1.08)
**Pre-pregnancy BMI (kg/m^2^)**		
Underweight (<18.5)	1.00 (Ref)	1.00 (Ref)
Normal (18.5–24.9)	0.87 (0.62–1.22)	1.37 (0.69–2.69)
Overweight/Obese (≥25.0)	0.63 (0.42–0.94)	1.13 (0.53–2.40)
**Sex of newborn**		
Female	1.00 (Ref)	1.00 (Ref)
Male	1.37 (1.10–1.69)	1.35 (0.92–1.97)
**Number of ANC visits**		
Inadequate <8	1.00 (Ref)	1.00 (Ref)
Adequate ≥8	0.64 (0.46–0.88)	0.75 (0.47–1.20)
**Number of SP-IPTp doses**		
None	1.00 (Ref)	1.00 (Ref)
Up to 2 doses	0.79 (0.57–1.09)	0.76 (0.44–1.31)
3 or more doses	0.65 (0.46–0.92)	0.53 (0.29–0.95)

Note: Multiple imputation (MI) was performed using chained equations (20 imputations; 5 iterations) to account for missingness in maternal/neonatal outcomes and select socio-demographic and clinical predictors. Complete-case analysis includes only participants with no missing values for all included variables.

**Table 9:** Comparison of factors associated with maternal anaemia at term using multiple imputation and complete-case analysis.

	Multiple Imputation (Pooled)	Complete-Case Analysis
	N = 5036	N = 1232
	Adjusted OR (95% CI)	Adjusted OR (95% CI)
**Region of residence**		
Ashanti region	1.00 (Ref)	1.00 (Ref)
Volta region	2.22 (1.78–2.75)	2.16 (1.62–2.88)
**Gestational age at booking (weeks)**		
1st Trimester (<14)	1.00 (Ref)	1.00 (Ref)
2nd Trimester (14–27)	1.12 (0.93–1.34)	1.03 (0.77–1.37)
3rd Trimester (≥28)	1.17 (0.78–1.75)	0.87 (0.45–1.71)
**Pre-pregnancy BMI (kg/m^2^)**		
Underweight (<18.5)	1.00 (Ref)	1.00 (Ref)
Normal (18.5–24.9)	0.91 (0.66–1.26)	0.75 (0.46–1.22)
Overweight/Obese (≥25.0)	0.86 (0.58–1.27)	0.73 (0.44–1.22)
**Number of ANC visits**		
Inadequate <8	1.00 (Ref)	1.00 (Ref)
Adequate ≥8	0.77 (0.63–0.94)	0.96 (0.70–1.30)
**Received ≥3 iron and folate doses**		
No	1.00 (Ref)	1.00 (Ref)
Yes	0.80 (0.66–0.96)	0.45 (0.32–0.64)
**Anaemia Status at Booking (g/dL)**		
Normal (≥11)	1.00 (Ref)	1.00 (Ref)
Anaemia (<11)	3.98 (3.31–4.79)	5.48 (4.20–7.15)

Note: Multiple imputation (MI) was performed using chained equations (20 imputations; 5 iterations) to account for missingness in maternal/neonatal outcomes and select socio-demographic and clinical predictors. Complete-case analysis includes only participants with no missing values for all included variables.

## Data Availability

The data presented in this study are available on request from the corresponding author. The data are not publicly available due to ongoing data analysis for subsequent publications.
